# Evaluation of Medicine Abuse Trends in Community Pharmacies: The Medicine Abuse Observatory (MAO) in a Region of Southern Europe

**DOI:** 10.3390/ijerph18157818

**Published:** 2021-07-23

**Authors:** Maria Perelló, Karla Rio-Aige, Rafel Guayta-Escolies, Pilar Gascón, Pilar Rius, Anna M. Jambrina, Guillermo Bagaria, Mercè Armelles, Francisco José Pérez-Cano, Manel Rabanal

**Affiliations:** 1 Barcelona College of Pharmacists, 08009 Barcelona, Spain; mperello@cofb.cat (M.P.); gbagaria001@cofb.cat (G.B.); 2 Physiology Section, Department of Biochemistry and Physiology, Faculty of Pharmacy and Food Science, University of Barcelona, 08028 Barcelona, Spain; karla22081996@gmail.com (K.R.-A.); amjambrina@gencat.cat (A.M.J.); mrabanal@gencat.cat (M.R.); 3 Institute of Research in Nutrition and Food Safety (INSA), 08921 Santa Coloma de Gramenet, Spain; 4 Blanquerna School of Health Sciences, Ramon Llull University, 08025 Barcelona, Spain; rafaelge@blanquerna.url.edu (R.G.-E.); mariapilargl@blanquerna.url.edu (P.G.); 5 Council of the Pharmacist’s Association of Catalonia, 08009 Barcelona, Spain; prius@ccfc.cat; 6 Directorate-General for Healthcare Planning and Regulation, Ministry of Health, Government of Catalonia, 08028 Barcelona, Spain; m.armelles@gencat.cat

**Keywords:** medicine abuse, community pharmacy, drug related disorders, non-medical use, pharmacist intervention

## Abstract

The misuse of medicines is a global public health concern that needs to be taken into consideration and requires actions across all government sectors and society. The aim of this study is to identify trends of drug abuse in Catalonia, a region of Spain located in the South of Europe. For this purpose, a questionnaire-based detection tool was created and implemented in 60 community pharmacies. Out of 548 questionnaires (98.4%), 64.2% of participants were men and the highest age proportion was 25–35 years (31.4%). Potential drug abuse was the highest in urban pharmacies (84.9%). The main drug class involved were benzodiazepines (31.8%), codeine (19.3%), tramadol (7.5%), methylphenidate (5.8%), gabapentinoids (5.8%), cycloplegic drops (4.4%), z-drugs (2.6%), piracetam (2.2%), dextromethorphan (1.6%) and clomethiazole (1.1%). The majority of drugs were requested without prescription (58.6%) and through probably forged prescriptions (23.7%). Slightly less than half (49.8%) of the patients request frequently to the pharmacist, especially in rural and mountain pharmacies (73.3% and 88.5%, respectively). A small proportion (10.8%) were requested with intimidation. Pharmacists only supplied in 21.7% of the cases. This study has demonstrated the suitability of the new detection system, being a useful approach to replicate in other locations with similar needs.

## 1. Introduction

Prescription drug abuse and misuse is defined as the intentional use of a medication without a prescription, in a way other than as prescribed, or for the experience or feeling that it causes [1]. However, also, the inappropriate use can be unintentional, such as when it is due to ignorance or cognitive impairment and is particularly common with elderly patients. Furthermore, addiction can result from taking medications and is characterised by persistent drug use or problematic behaviours, despite knowledge of the negative consequences [2].

Medicine misuse is a broad term, which includes many different types of problematic consumption and has been increasing in the past few years. According to the National Survey on Drug Use and Health (NSDUH) for national indicators of substance use and mental health among people aged 12 or older in the civilian, an estimated 16.9 million Americans (6.2% of population) aged 12 or older misused prescription psychotherapeutic drugs at least once in 2017. This number included 9.9 million who misused prescription pain relievers in that period, 5.1 million who misused prescription stimulants and ~6.4 million who misused prescription tranquilisers or sedatives [3]. In addition, the Drug Abuse Warning Network (DAWN) is a nationally represented public health surveillance system that continuously monitors drug-related visits to hospital emergency departments.

Currently, there are state programmes, prescription drug monitoring programs (PDMP), which are nationwide electronic databases that collect data on substances dispensed in the state [4]. They are tools used by states to address prescription drug abuse, addiction and diversion, which may be adequate for several purposes such as to identify and to prevent drug abuse and diversion and to inform public health initiatives through outlining of use and abuse trends.

In Europe, the European Drug Emergencies Network (Euro-DEN Plus) monitors drug-related emergency presentations across Europe to provide unique insight into acute health harms related to drug use [5]. A total of 29 sentinel centres in 21 European countries set up the network, supported by the EU drugs agency (EMCDDA). Of the 23,947 presentations in the 4-year Euro-DEN Plus dataset, 6207 (25.9%) involved at least one prescription medicine, and of these almost half (2876; 46.3%) involved only prescription medicines, with no other established illicit/recreational drug or NPS. The mean age of people with emergency presentations related to toxicity, in which only prescription medicines were involved, were slightly older (mean age 34 years, IQR 28–43 years) than those people with overall presentations (mean age 31 years, IQR 25–39 years). The proportion of women was higher (31.6% in the prescription-only group, compared with 23.8% for all Euro-DEN Plus presentations) [6].

This available information focuses at European-level instead of at individual member states and a surveillance system that estimates the prescription drug abuse and misuse on the general population does not exist. As the misuse of medicines in Europe is an increasing issue of concern, investigations are required to monitor prescription drug abuse, to identify its scope and develop targeted interventions [7].

In Spain, the Spanish Household Survey on Alcohol and Drugs (EDADES) is used in research on consumption of addictive substances to understand the situation and trends relating to drug use in the population living in Spain in order to obtain useful information to design and evaluate policies aimed at preventing drug use and associated problems [8].

In addition, the Secondary School Students Survey on Drugs (ESTUDES) allows obtaining information among secondary school students. In both, hypnosedatives and opioid analgesics are the only medicines the surveys ask for. There is no other source that provides information on consumption of these or other prescription or non-prescription drugs. The results show that in Spain, in 2017, 7.5% of people aged 15–64 took hypnosedatives in the last month and the greatest proportion (80%) were daily users. In addition, the highest proportion of students uses hypnosedatives after alcohol, tobacco and cannabis. As for opioid analgesics, 14.5% of the population admitted consuming opioid analgesics occasionally and 2.2% of the students used them to get high, at least once in their life [9].

Community pharmacists are the most accessible health professionals to the public. To ensure an accurate supply of appropriate products, their professional activities also cover counselling patients at the time of dispensing prescription and non-prescription drugs, giving drug information to health professionals, patients and the public, and participating in health-promotion programmes. They maintain links with other health professionals in primary health care. Pharmacists have comprehensive knowledge about the safe and effective use of medications and about the adverse effects of their inappropriate use [10]. To manage medicine abuse and misuse, interventions at different levels are needed, including the support of electronic records initiatives, such as a pharmacy network to communicate relevant information about medicines use and to refer patients with medicine-related issues. Due to this task, common practices among medicine abusers, such as counterfeit prescriptions and doctor shopping, can be reduced [11].

The aim of this study is to identify trends of medicine abuse in Catalonia, a region of Spain located in the South of Europe. For this purpose, we aim to create a new detection tool based on a questionnaire through community pharmacies in Catalonia and its implementation in a surveillance system to obtain and analyse these data that could be applicable to other scenarios and geography.

## 2. Materials and Methods

### 2.1. Medicine Abuse Observatory (MAO) Conceptualisation

As a first step, with the aim to act on the medicines abuse phenomenon, it is necessary to create an epidemiological surveillance system. For that, the Medicine Abuse Observatory (MAO) is created as a project supported by the Catalonia Pharmacists Council and the Ministry of Health of the Government of Catalonia. Intermediate goals are to establish a strategic framework, criteria for their functionalities and operating procedures. Specific aims are to analyse the trends of medicines abuse and misuse of the population in Catalonia through community pharmacies, to define guidelines, to collaborate with other health professionals and to consolidate the pharmaceutical role as a health public agent.

For this purpose, a systematic search was made in several online sources to explore already existing tools. Specifically, a PubMed search was conducted in January 2017 using the following keywords: prescription drug abuse, community pharmacy and substance-related disorders. We found 836 articles corresponding to the 2004–2017 period from which only 25 provided similar approaches that could help design our questionnaire –community pharmacy involvement as a main axis for data collection or its participation in the network with other healthcare systems. For similar purposes, we sought information from different organisations such as NIDA, WHO and EMCDDA and to provide data and trends from other countries that could also help collecting data for questionnaire design.

All these publications with a particular role of community pharmacy included a wide-ranging methodologies and instruments in this area, including specific questionnaires to monitor a particular drug or general surveys of forged prescriptions to investigate the risk of psychoactive medications abuse. Considering the above, we developed a novel questionnaire-based screening tool to collect data regarding medicine abuse and misuse and to analyse the trends and eventually provide a basis for epidemiologic research.

### 2.2. Abuse Drugs Questionnaire (ADQ) Development

As it is difficult to recognise abuse or misuse because information provided by the patient is usually subtle and not strongly justified, we base on an approach proposed by Finch in 1993, which provides signs and behaviours that give a clearer indication that a drug abuse problem exists [12]. These elements are as follows: (i) Pattern of calling for refills after hours and/or repeatedly needing early refills. (ii) Prescriptions from multiple physicians. (iii) Frequent visits to emergency rooms. (iv) Strong preference and knowledge for a particular drug. (v) Incongruence between severity of the complaint and the physical presentation.

This theoretical framework provides guidance in the design and implementation of the intervention, as it can specify the relation between constructs and the determinants of people who present drug-seeking behaviour. Therefore, the elements presented above have been used to create a Abuse Drug Questionnaire (ADQ). For this reason, and considering the situations that would arise in the community pharmacy, the questions included in the ADQ are the request way of medication, make a frequent demand of the substance, and also intimidate. The substances to follow up are chosen taking into account the evidence from scientific literature and data from our environment. As there was not a standardised questionnaire, previously to the final ADQ, a pilot study was carried out in 2016 in 21 community pharmacies in Barcelona, Catalonia, Spain, with the aim to validate the ADQ. The study lasted 6 months and allowed us to collect 49 notifications of suspected drug abuse. With this data, the final ADQ is an anonymous multichoice test containing 10 closed and two open-ended questions, categorised in four different parts (Table 1). In questions 1 and 2, pharmacist identification is required; in questions 3 to 5, patient demographic variables as age, sex and origin are included. Questions 6 to 9 are intended to exactly specify the substance involved (in case of benzodiazepines specifying which from a list), and how it is requested. In this sense, it is considered “does not require a prescription” for over the counter (OTC) medicines and “requested with prescription” for prescriptions needing frequent refills and/or from multiple physicians. We consider “probably forged prescription” as a counterfeit prescription (copies) or any falsification made on a right prescription form.

Regarding substance abuse, the ADQ is structured in two scopes, medicines that can be abused (used for therapeutic purpose but inappropriate patterns) and medicines that can be misused (usually used to modify mood or to have psychedelic experiences). In addition of chosen substances, it is also included an item entitled “others” that allows pharmacists to report any medicine. In general, the most involved prescription drugs are opioid analgesics, central nervous system (CNS) depressants (sedatives, tranquilisers and hypnotics) and stimulants (generally used to treat attention-deficit hyperactivity disorder (ADHD) and narcolepsy). Among OTC medicines, dextromethorphan (a cough suppressant) is frequently used. Finally, in questions 10 to 12, pharmacist management is enquired. The aim of these three questions is to know in which cases the medicines are dispensed and to study the reasons for which these medicines are dispensed. The pharmacist filled out the questionnaire when a patient that requested a medicine presented two or more of the defined signs and behavioural symptoms and was suspected of being a medicine abuser [12]. Patient information was obtained anonymously by observation during the interview and neither verbal nor written consent were needed.

A web-based survey and data collection software, easy to use for questionnaire-based closed and open-ended questions, called Typeform (Typeform SL, Barcelona, Spain) is used. The pharmacist acceded to the ADQ through a link embedded into the Barcelona College of Pharmacists (COFB) website, the principal online work tool for pharmacists. This software allowed obtaining the ADQ electronic data from respondents and transforming the collected data to excel, thus facilitating continuous statistical operations and analysis.

### 2.3. Pharmacies Selection

The study was conducted between July 2017 and December 2019 in 60 community pharmacies scattered throughout Catalonia. The pharmacies included constitute a proportional stratification of the population in Catalonia based on Spain sentinel model and criteria of representativeness and ensuring coverage of 2.5% of the Catalan population [13].

For that, the basic health areas (BHA) of each of the healthcare sectors of Catalonia were used. In Catalonia, in accordance with the law of pharmaceutical regulation, there are three types of BHA: the urban BHA (U), whose territorial delimitation is included in a single municipal term or 90% of the population resides in the same municipal term; mountain BHA (M), which include only mountain or high mountain areas, defined by regional regulations; and the rural BHA (R), which are those not included in the other two groups.

In this context, the proportion of urban (U), rural (R) and mountain (M) pharmacies in each Catalan BHA was established, considering socioeconomic, demographic and epidemiological factors.

Once the area to be studied was selected, the willingness of pharmacies to participate in the study was required and after written form was obtained from the pharmacy managers to take part in this study.

### 2.4. Pharmacist Training

To facilitate information exchange, the management of data and the training sessions aimed at pharmacists were carried out by the Drug Information Centre (DIC) of the COFB, who validated the notification and treated the data in order to create a database and make reports to transfer to the stakeholders. Pharmacists were trained at multiple times during the study to minimise decay of skills and resolve questions and problems that have been identified. The training programme consisted of a one-hour-and-a-half interactive instruction, and it mainly addressed capacity and skills development. The scheme of the session contained three parts: (i) Theoretical framework: concepts, prevalence, trends and world data. (ii) Basis of the method: Finch criteria to identify behaviour related to potential drug abuser. (iii) Operational procedures: dispensing service, patient interview, identifying signs and online form comprehension. Furthermore, feasible patterns were shown. Reinforcement training sessions were conducted every 6 months to resolve issues and clarify questions about screening procedures.

### 2.5. Statistical Analysis

Patient’s characteristics that were categorical variables were summarised as counts and percentages. Continuous variables were summarised as means with standard deviations. For the statistical analysis, the χ^2^ test was used for the study of the categorical variables. A *p* value < 0.05 was considered statistically significant. All analyses were conducted with SPSSS software, version 18 (SPSS Inc., Chicago, IL, USA).

Moreover, clustering of the study groups was analysed by non-metric multidimensional scaling (NMDS) in Rstudio using the R package vegan (Community Ecology Package. R package version 2.4-6). In the NMDS plot it was represented the complex dimensional data in two dimensions to highlight the similarities between customers in terms of their characteristics and behaviour. In this term, the further two samples are from each other, the less similar they are. Furthermore, more information was overlaid on ordination NMDS plot with the function “envfit” to represent vectors onto the plot. Longer vectors mean a stronger association with the participants in that direction.

## 3. Results

Out of 557 questionnaires received, 548 were included in the study (98.4%). Nine questionnaires were excluded as they did not considered cases according to the inclusion criteria or the pharmacist did not provide information.

### 3.1. Patient and Pharmacy Distribution Profile

**Figure 1:**
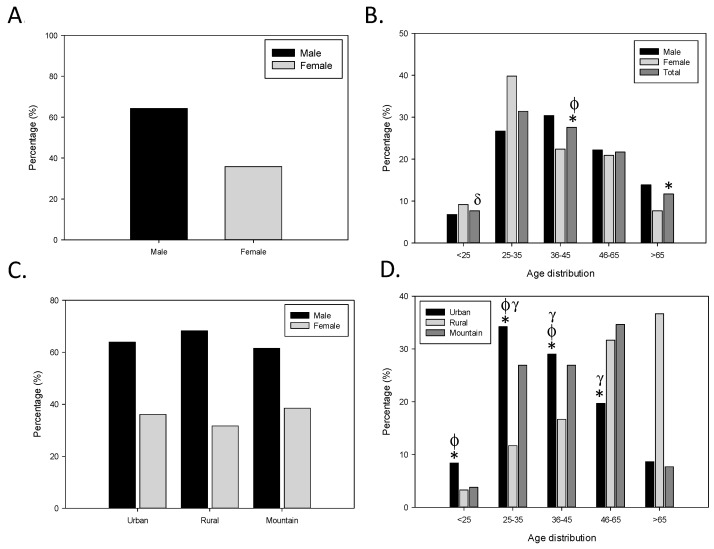
Patient and pharmacy distribution profile. Distribution of participants according to (**A**) sex, (**B**) age and sex, (**C**) type of pharmacy and sex and (**D**) type of pharmacy and age of the participants. The proportions are calculated from each condition related to 548 notifications analysed. Patient distribution profile was not equally distributed among sexes, age or type of pharmacies. These differences were even more clear when the age and type of pharmacy analysis was performed together. Statistical differences: (**B**) * *p* < 0.05 vs. range 25–35, ɸ *p* < 0.05 vs. range < 25, ^δ^ *p* < 0.05 vs. range > 65 and (**D**) * *p* < 0.05 U/R in range > 65, ɸ *p* < 0.05 U/R vs. range 46–65, ^γ^ *p* < 0.05 R/M in range > 65.

Patient distribution profile was not equally distributed among sexes and ages. Regarding the sex of the patients reporting drug abuse, they were mostly men (64.2%) (Figure 1A), and when the age of the medicine abuser was considered, the highest proportion was found in the age interval of 25–35 years (31.4%), followed by 36–45 years (27.6%), 45–65 years (21.7%) and >65 years (11.7%). The lowest proportion of prescription drug users was found in the youngest interval of age considered (<25 years) (Figure 1B). The combination of both data allows to better characterise the profile of the patients showing a differential predominance pattern between men and women. In the most prevalent age range of medicine abusers (25–35 years), there are more women than men included (54.65 vs. 45.35, respectively, *p* < 0.05); however, this pattern is inverted later one and the proportion of men vs. women increases up to the highest difference in the oldest group (76.56 vs. 23.44, respectively, *p* < 0.05).

The number of reported cases of potential drug abuse was the highest in urban pharmacies (U), followed by rural (R) and finally mountain (M) pharmacies (462, 60 and 26 notifications, respectively). Thus, urban pharmacies represent 84.91% of the cases, accounting then the rural and the mountain pharmacies with a very low proportion (10.95% and 4.74%, respectively). In addition, the medicine abuser profile changes in terms of age but not sex depending on the pharmacy location. The proportion of males/females were similar in all three types of pharmacies: 63.9/36.1 (U), 68.3/31.7 (R) and 61.5/38.5 (M) (Figure 1C). However, age distribution (Figure 1D) showed that according to the type of pharmacy and discarding the youngest group (<25 years), the proportion of the detected cases in urban pharmacies decreased with age, whereas those in rural pharmacies increased with age. Indeed, the youngest patients (25–35 years) were more likely linked to urban pharmacies (34.2%; *p* < 0.05) and the oldest patients (>65 years) to rural pharmacies (36.7%; *p* < 0.05). The age proportion distribution in mountain pharmacies is similar between 25–65 years (26.9–34.6%) and dropped in >65 years to 7.7% of the cases (Figure 1D).

Another important variable was considered—whether the notifications belonged to native or non-native patients in the pharmacy. Overall, an approximately 60/40 proportion was found for native/non-native patients considering the three types of pharmacies together. However, this proportion was significantly different in U than R or M pharmacies and also between sexes (*p* < 0.05). Specifically, urban pharmacies notifications involved a high proportion of non-native patients (45.5%) when compared to rural and mountain pharmacies, in which less proportion of notifications from non-natives were recorded (23.3% and 15.4%, respectively; *p* < 0.05). The pattern of men and women was totally different in this aspect, as zero cases of non-native women were detected. Focusing then on the notifications from men in all types of pharmacies, we can observe that, as in the overall population, the proportion of cases from non-natives is the highest in urban pharmacies, although this proportion has even increased (71.2%), whereas a 65–70% proportion is found in the mountain and rural pharmacies for the native patients.

### 3.2. Involved Substances

**Figure 2:**
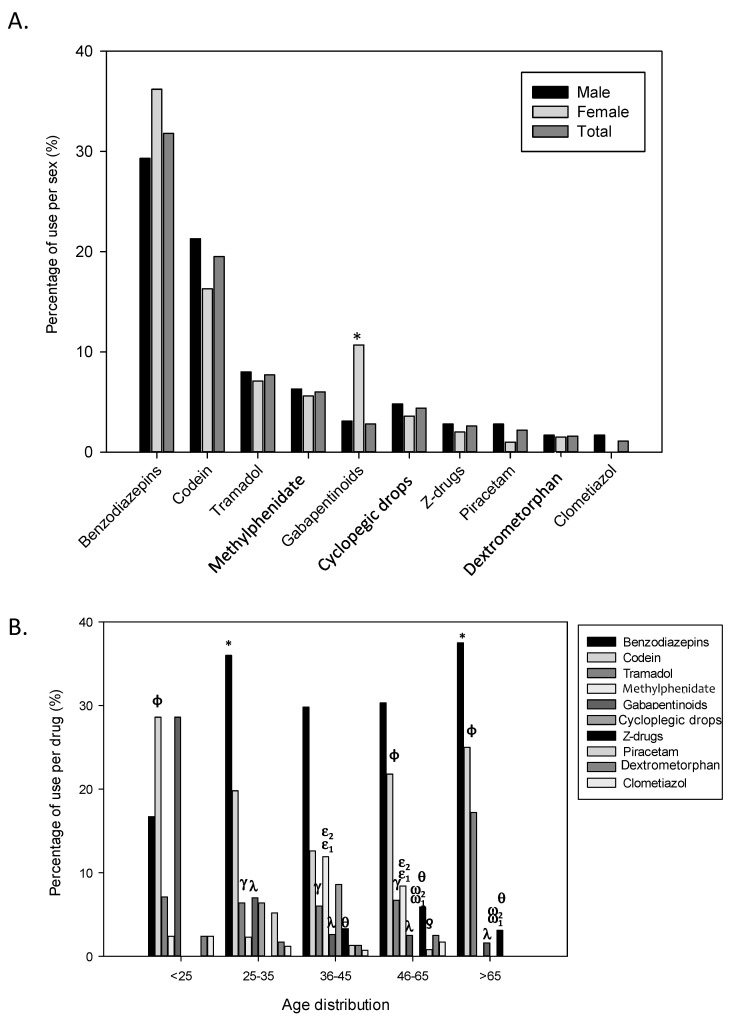
Proportion of the type of drug. Distribution of the participants according to (**A**) type of drug and sex and, (**B**) type of drug and age. The proportions are calculated from each condition related to 548 notifications analysed. As it can be observed the drug abuse was also differentially distributed by age and different patterns were obtained for specific drugs and ages. Statistical differences: (**A**) * *p* < 0.05 vs. men and (**B**) * *p* < 0,05 vs. range < 25 in benzodiazepins group, ɸ *p* < 0.05 vs. range 36–45 in codein grup, ^γ^ *p* < 0.05 vs. range > 65 in tramadol group, ^ε1^ *p* < 0.05 vs. range 25–35; ^ε2^ *p* < 0.05 vs. range > 65 in methylphenidate group, ^λ^ *p* < 0.05 vs. range < 25 in gaba group, ^ω1^ *p* < 0.05 vs. range 25–35; ^ω2^ *p* < 0.05 vs. range 36–45 in cicloplegic, ^θ^ *p* < 0,05 vs. range 25–35 in z-drug group, ^ƍ^ *p* < 0.05 vs. range 25–35 in piracetam group.

Substances involved in a possible medicine abuse demand in the pharmacy are quite variable (Figure 2). Considering all the notifications obtained, the main drug class involved were benzodiazepines (31.8%), followed by codeine (19.3%), tramadol (7.5%) and methylphenidate (5.8%). Gabapentinoids were demanded by 5.8%, cycloplegic drops by 4.4%, and z-drugs by 2.6%. Additionally, other medicines required in lower proportions were piracetam (2.2%), dextromethorphan (1.6%) and clomethiazole (1.1%) (Figure 2A). Finally, a miscellany of minority substances (<1%) such as laxatives, analgesics, antidepressants, decongestants, antibiotics, diuretics, antidiabetics or beta-adrenergics were also detected.

Considering the medications that require prescription, the patient presented a prescription in 35.7% of z-drugs requests, 21.8% of benzodiazepines, 16.7% of clometiazol, 14.6% of tramadol, 4.7% of codeine and 3.1% of gabapentinoids. All the cases of methylphenidate, cyclopegic drops and dextromethorphan correspond to demands made to extort the drug, both requesting without prescription or through a counterfeit one. 

Behaviour regarding to sex was similar in all the substances (Figure 2A), and only one difference raised when considering the drug abuse of the gabapentinoids in which the females used them (10.7%) around 3 times more than the males (3.1%, *p* < 0.05). 

Drug abuse was also differentially distributed by age (Figure 2B), being benzodiazepines followed by codeine the most consumed drugs in all age groups >25 years. This pattern was different in younger consumers (<25 years), in which the predominant drug was codeine and, benzodiazepines were the third drug in importance because gabapentinoids were highly detected in ˂25 years (28.6%) compared with the rest of age groups (<7% in all cases, *p* ˂ 0.05). As for tramadol, the highest consumption was detected in the oldest group. As for methylphenidate, the highest consumption was detected in the 36–45 age group (11.9%) and no cases were observed in patients ˃ 65 years. Only patients aged 25–45 were associated with the use of cycloplegic drops. Z-drugs were identified in older groups (>35 years) being no differences between them. The drug abuse of piracetam, dextromethorphan and clomethiazole had similar proportions in all age groups. When age and sex data analysis were combined, no significant differences appeared. Although some tendencies were observed when the type of pharmacy was also included no significant differences were detected; as in the case of the higher benzodiazepine use in urban and rural pharmacies (31.6% and 36.7%, respectively) than in mountain pharmacies (23.1%).

### 3.3. Drug Request

**Figure 3:**
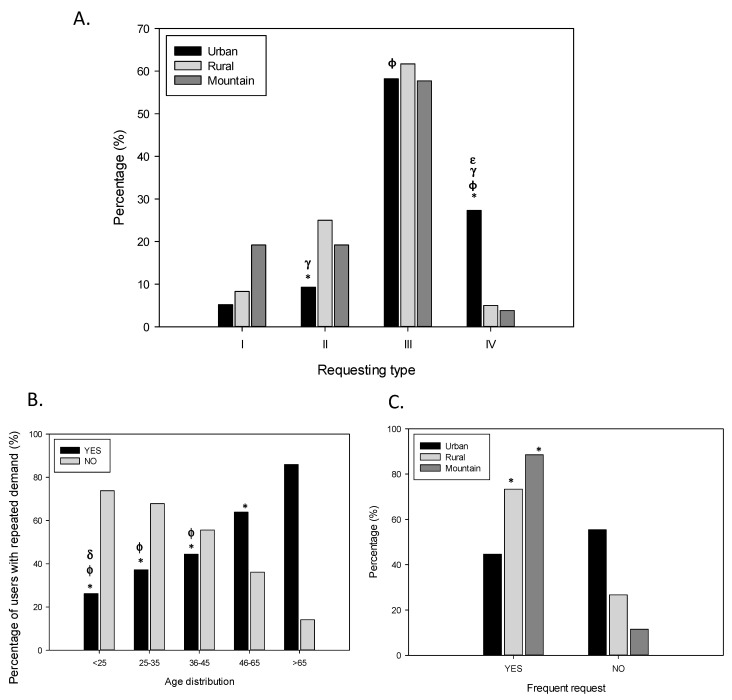
Drug-requesting behaviour of patients. Distribution of the participants according to (**A**) type of request and type of pharmacy, (**B**) repeated demand and age, and (**C**) frequent request and type of pharmacy. Requesting types are as follows: I, prescriptions corresponding to an over the counter (OTC) drug; II, formal prescription; III, without prescription; and finally, IV, forged prescriptions. It can be observed that the drug request behaviour depends on the type of pharmacy, the repeated demand depends on age and that the frequent request is produced at higher levels in rural or mountain pharmacies. proportions are calculated from each condition related to 548 notifications analysed. Statistical differences: (**A**) * *p* < 0.05 U/R vs. I type, ɸ *p* < 0.05 U/M vs. I type, ^γ^ *p* < 0.05 U/R vs. III type, ^ε^ *p* < 0.05 U/M vs. III type, (**B**) * *p* < 0.05 vs. range >65, ɸ *p* < 0.05 vs. range 46–65, ^δ^ *p* < 0.05 vs. range 36–45, and (**C**) * *p* < 0.05 vs. U.

Drug request was also considered (Figure 3). Drugs were most commonly requested without prescription (58.6%, Type III) followed by a probably forged prescription (23.7%, Type IV). Moreover, in 11.5% of the answers, the drug was requested with a formal prescription (Type II) and in 6.2% of the cases the substance corresponded to an OTC (Type I). No differences were detected regarding sex. These proportions were similar among all the age groups studied, with exception of the fact that patients aged ˃ 65 were not associated with falsified prescriptions in any case (0%, *p* ˂ 0.05). The percentage of drug request without prescription was also similar when considering the type of pharmacy and only the detection in the use of forged prescriptions was found the highest in urban pharmacies (27.3% vs. 5% and 3.8% for U, R and M, respectively, *p* ˂ 0.05) (Figure 3A).

Repeated requests for drugs are sometimes made to try to get the substance, and in fact 49.8% of the abusers admitted it. However, this behaviour is age-dependent and the older groups were more involved in making repeated demands. This can be observed when comparing the patient proportion in the group aged ˃65 (85.9%) with the group aged ˂ 25 (26.2%) (*p* ˂ 0.05, Figure 3B). No differences between sexes were observed. Finally, repeated demands occurred more frequently in rural and mountain pharmacies (73.3% and 88.5%, respectively) than in urban pharmacies (44.6%) (*p* ˂ 0.05, Figure 3C).

In addition, in some cases (10.8%) drug requests occurred with verbal intimidation. This aspect was not significantly related to the sex, being their proportions 57.6% and 42.4%, respectively. Moreover, in this line, the proportion of intimidating patients were not either associated to age. However, this behaviour was more frequent in mountain pharmacies (23%) with respect to urban pharmacies (9.3%) (*p* ˂ 0.05).

### 3.4. Supply of Medicine

**Figure 4:**
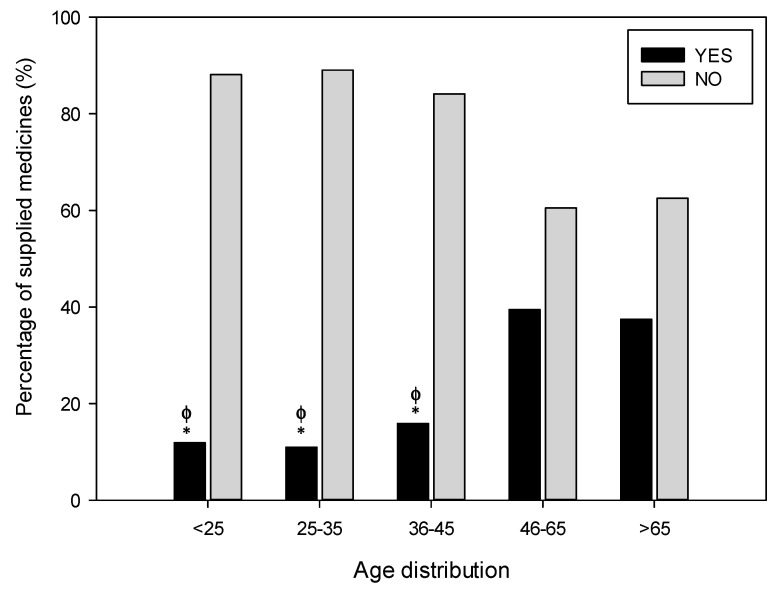
Distribution of the supplying behaviour according to the age of the abuser. The proportions are calculated from each condition related to 548 notifications analysed. It can be observed that the pharmacist supplies the request drug more frequently at older ages. Statistical differences: * *p* < 0.05 vs. range >65, ɸ *p* < 0.05 vs. range 46–65.

Due to all the above features (forged prescription, intimidation, etc.) in 78.3% of the cases the drug was not supplied to the requesting medicine abuser by the pharmacist. Overall, the sex, age and type of pharmacy were influencing factors. On the one hand, the drug was supplied to 25.9% of the requesting men whether this proportion was significantly lower for women (14.3%, *p* < 0.05). Regarding the age, older groups got the substance more often than younger groups, with the age group of >65 years and 46–65 years being those getting the drug in the highest proportion (37.5% and 39.5%, respectively) and patients aged < 25 with the lowest proportion (11.9%, *p* ˂ 0.05) (Figure 4). Finally, regarding pharmacy type, rural and mountain pharmacies supplied the medicine more frequently (35% and 46.2%, respectively) than urban pharmacies (18.6%, *p* ˂ 0.05).

It has to be taken into account that 21.7% of the pharmacists declared to supply the drug. The main reasons given by the pharmacists for dispensing a drug were as follows:

i. the pharmacist knows the patient because that patient lives in the neighbourhood;
ii. the requested medication is an over-the-counter product;
iii. the pharmacist has detected that the prescription was false after dispensing the medication;
iv. the patient presents multiple prescriptions but all of them are correct;
v. the patient knows why and how the medicine is used and also expresses a need and the pharmacist gives the medication under pressure.

Referring to the remarks section, regardless of whether or not the dispensation was made, the pharmacists commented on this:

i. the patient take more medication than prescribed;
ii. the reason for the demand does not agree with the symptoms described;
iii. it has been confirmed with the doctor that the prescription is false;
iv. the patients insists forcefully for getting the drug;
v. a first dispensation is made but then the following are denied.

### 3.5. Multidimensional Analysis of the Participants

**Figure 5:**
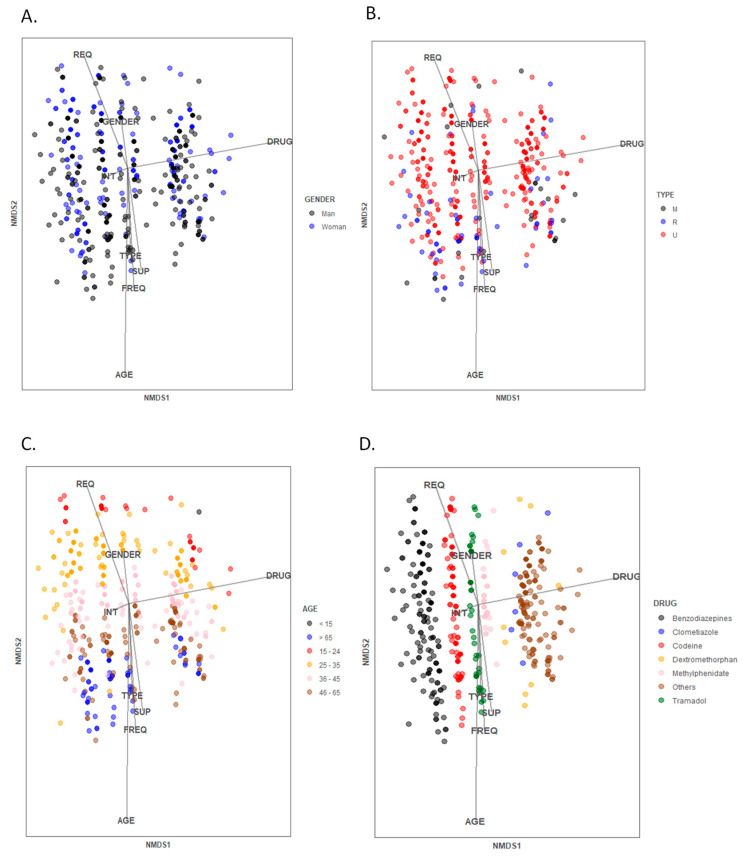
Distribution of the studied population analysed by non-metric multidimensional scaling (NMDS). Different influencing characteristics of the participants are depicted in colours: (**A**) gender, (**B**) type of pharmacy, (**C**) age and (**D**) type of drug requested. The vectors correspond to the variables analysed together: GENDER, DRUG, TYPE (type of pharmacy), SUP (whether the drug is supplied or not), INT (request with intimidation), FREQ (frequency demanding the drug) and AGE. The analysis includes the 548 notifications. It can be observed the clustering of the users depending on the stablished parameters being the age and type of drug the clearest influent factors.

After evaluating the influence of several participant features individually or combining them, a multiparametric approach allows an overall observation of the main aspects involved in drug abuse behaviour and their relationship (Figure 5). The main influencing factors involved were the type of drug, age of the patient and type of request as the vectors obtained in the NMDS analysis have the highest module. From this initial evaluation it was observed that neither the gender (Figure 5A) nor the type of pharmacy (Figure 5B) led to a clear cluster distribution. However, the age (Figure 5C) and type of drug (Figure 5D) were determinant factors for clustering the medicine abuser characteristics.

## 4. Discussion

The misuse of medicines is a global public health concern that needs to be taken into consideration and requires actions across all government sectors and society. Pharmacists, as healthcare providers, should be actively involved in reducing the negative impacts of substance abuse on society, health systems and the pharmacy profession [10]. Epidemiological surveillance systems represent the best strategy to address these objectives, especially when implemented where the problem is expressed. Furthermore, pharmacists have the knowledge about the safe and effective use of medications and about the adverse effects of their inappropriate use so they can act in substance abuse prevention and education [11,14,15].

The Health Plan for Catalonia 2016–2020 aims toward integrated care that seeks to deal with medical complexity and interrelate with the person’s environment. A work pillar is to consolidate the function of the pharmacist as a healthcare educator, to provide information about medicines and to promote the portfolio of services. In this context, this study proposes a screening to detect medicine abuse and misuse, validate its usefulness and suitability and describe the associated factors. Over the last years, some articles have been published regarding aspects of prescription and non-prescription diversion but, as far as we know, most of the data come from case reports or observations from epidemiological studies [7]. For example, the Drug Abuse Warning Network (DAWN) monitors drug-related hospital emergencies and drug-related deaths. In addition, the European Monitoring Centre for Drugs and Drug Addiction (EMCDDA) collects, analyses and disseminates scientifically information on drugs and their consequences based on the latest findings on drug-related hospital emergencies from a network of sentinel hospitals across Europe [6,16].

Besides, some studies describe the role of community pharmacy on the management of prescription and non-prescription drug abuse phenomenon from different frameworks as surveys or reviews that allow showing their ability to detect drug-related problems [17,18,19,20,21,22,23,24,25,26,27,28,29,30]. However, none of them focused on all available medications and ways to try to get them from the pharmacy, which include patient behaviour. As no standard tool was available, a specific questionnaire was designed for this aim in the present approach. The results from this questionnaire allowed detecting any drug abuse trends in a large sample of the population providing high positive predictive value due to the sensitivity of the tool used. In addition, this procedure is simple and robust to detect cases due to its usability and integration into the working tool used by pharmacists in their daily practice. The results obtained allowed us to observe the factors influencing this process in terms of sex, age, pharmacy type, patient origin and behaviour, among others.

First of all, our study did not allow us to observe differences according to sex. In fact, current evidence on sex differences in health is limited, but in general men are more likely than women to use almost all types of illicit drugs, including misuse of prescription drugs. However, some research has observed that women are more likely to have chronic pain and to take prescription opioids to cope with pain as well as other problems, such as anxiety or tension [31].

Second, and related to age, we have observed a different pattern not only associated to the type of drug, but also to the behaviour associated in requesting the drug and in the supplying success. Besides particular drug relationships, these results are overall in agreement with the current literature evaluating prescription fraud and abuse of drugs used for self-medication, which show a mean age of 45 years approximately. Despite this, substances such as methylphenidate or cycloplegic drops are associated with use at a younger age.

In addition, one of the main outcomes was to provide evidence about the influence of the location of the pharmacies in some aspects related to the drug abuse. Our results showed a differential behaviour in the patient in smaller mountain or rural pharmacies when compared to urban ones. In fact, our data allowed us to observe that urban pharmacies are related to less regular patients, enabling greater anonymity, both for the patient and the pharmacist, as suggested by others [21,22,23].

Taking all these factors together and focusing on patient behaviour, in this study, in rural and mountain pharmacies it is observed that the older the population, the more repeated the request of the substance and, at the same time, the more it is supplied, especially in men. From these findings, we should be aware of the habits of small geographical areas where people know each other better and it is more difficult to challenge patients. Equally, this fact is also noticed in the use of bullying in mountain pharmacies. In contrast, urban pharmacies have a younger patient profile as well as a higher use of counterfeit prescriptions.

Regarding the substance asked for the drug abuse, data collected are similar in trends and types of substance abuse as data identified in other studies [32,33]. In particular, the most common substances are central nervous system depressants, opioids and stimulants. In addition, the use of certain medications such as cycloplegic drops, gabapentinoids, laxatives, antihistamines, analgesics, antidepressants and decongestants was also detected. In this article, we have focused on drugs that were reported to be more than 1%.

Benzodiazepines are the most notified substances in all age groups except for those aged under 25. As well, z-drugs are identified and particularly linked to an elderly patient profile. This fact could reflect that the utilisation of benzodiazepines and z-drugs has substantially increased with the ageing of the population, and they are most often prescribed in general practice, and it is well described that they are addictive and often used in larger doses and for a longer time than recommended [29,34,35]. In our approach, it was detected that more than half of benzodiazepines reports indicated that the patient made repeated requests of the substance through a forged prescription. This behaviour was especially related to urban pharmacies, whereas in rural and mountain pharmacies the request was frequently made without prescription. This is the first study showing these differential patterns associated to the type of pharmacy.

Codeine is the second substance more involved and 78.3% of the requested participants in the present study tried to get it without prescription although it is necessary. In the European Union (EU), codeine-containing medicines have been approved via national procedures and are available either on prescription (Spain) or as OTC in the different Member States, which must be taken into account in its availability [36]. The results suggest that all groups of age are identified in the codeine abuse, without differences among them, so a unique profile cannot be described. Codeine is used for self-medication as a pain reliever but also can be used as an illicit drug as Orriols et al. reported in a pilot study based on community pharmacies conducted in France [30].

Tramadol is mainly requested without prescription and half of the cases made repeated requests over time. Rural pharmacies reported more cases, and although it was frequently associated to patients aged > 65, there are not significant differences between age groups. Roussin et al. reported that problematic uses are also encountered among older subjects, within the setting of a medical treatment in order to avoid a withdrawal syndrome after discontinuing tramadol. However, it is suggested that the purpose could be also to obtain a particular effect [37,38]. Recently, the Euro-DEN Plus also evaluated tramadol misuse and concluded that although is low in frequency, admissions related to tramadol misuse can be a significant burden on emergency departments [39].

Methylphenidate had a high demand with falsification prescriptions (78%), mostly associated to urban pharmacies in the younger population. These results are in line with others shoeing that methylphenidate displayed the highest reports of falsification, particularly in Spain [29,40,41].

The use of gabapentinoids is associated to young women patients and mainly in the urban environment. This pattern is in accordance with some data about the detection of pregabalin abuse in urban emergency departments [42]. Moreover, in most of the reports (93.8%), the request was made without prescription, which is in line with the possible recreational use or to people with a history of substance abuse as described elsewhere [43,44]. A recent study points to a higher prevalence of lifetime chronic pain, lifetime illicit drug use and previous substance abuse treatment in those who non-medically use GABA analogues [45].

From this study, it can be observed that middle-aged men were identified as the most implicated group in demanding cycloplegic drops, which coincided with some studies that had also identified this pattern as well as VigiBase, a global database of individual case safety reports [46,47]. These last sources showed that cycloplegic drops can be used to get different effects as to attenuate opiate withdrawal symptoms and to enhance heroin effects.

Dextromethorphan is a popular OTC drug that when misused can lead to serious symptoms, including hallucinations. This recreational use has been reported in several countries [24,30]. The present study allows to observe that dextromethorphan has been involved in all groups of age, except in >65 years, and more than half (55.6%) of the requests were supplied by pharmacists because they did not require a prescription although they could identify abuse signals from the patient. This fact reveals the need for further education for community pharmacists in the management of patients at risk of medication misuse and diversion.

It is remarkable that more than half (58.6%) of the total reports are concerned with requests made without prescription although it was required by the drug. Moreover, 23.7% of them were made using a forged prescription, which is a critical outcome, even more so when considering an electronic prescription system in the studied area. Therefore, it could be appropriate to frame a network of community pharmacies for the purposes of monitoring prescription drug abuse in the area of public health and indirectly to confirm that electronic prescribing will limit prescription fraud [29]. Other systems implemented also allow knowing new patterns of drug diversion, as the collection of suspicious prescriptions through a systematic periodic survey, as it is the approach carried out in France [28].

Overall, intimidation behaviours are particularly associated with the demand of benzodiazepines in mountain pharmacies, which are more isolated and maybe patients have harder conditions to visit a doctor and receive a follow-up treatment. Pharmacists supplied the medicine more frequently in older patients from mountain pharmacies, as it is expected because of the characteristics in this area. In observations, pharmacists explained whatever they observed and considered relevant, for example, when a patient was observed that practice doctor-shopping or had a suspicious behaviour, but also described when a counselling was made and when a forged or false prescription was identified and verified with the centre. These comments provide extra useful information.

Despite this, the present approach has some limitations. The presented data can be used to determine certain regularities, but they are not sufficiently confirmed. To date, it is difficult to identify some behaviours, for example, those associated to drug requests because of the trouble to visit a doctor for refills, and due to some subjectivity in the detection of behaviours suspected of misuse and abuse. Furthermore, the difficulty in achieving exhaustive detection of falsified prescriptions as there is no gold standard. These facts could lead to an underestimation of the obtained results. However, it must be taken into account that the main aim of the study was to identify trends about the most diverted drugs and set a surveillance system in the community pharmacy. The implementation of this innovative pharmacy programme requires coordination between the different organisations and good skills of communication with the participants.

## 5. Conclusions

In conclusion, the present study has demonstrated the suitability of a new detection questionnaire and system through the community pharmacies in Catalonia. It has been appropriate at both organisational and operational levels for detecting the trends of drug abuse, being a useful approach to replicate in other locations with similar needs. This study highlights that spread out community pharmacies throughout mountain, rural and urban areas enable day-to-day contact and service of the entire population, which facilitates the potential role of a pharmacy network in addressing prescription drug abuse.

## Figures and Tables

**Table 1:** Abuse Drug Questionnaire (ADQ) used for the surveillance system implementation.

Question Number	Question Text	Answer
1	Pharmacy ID	
2	Pharmacist	
3	Patient age	<25/25–25/36–45/46–65/> 65
4	Patient sex	Male/Female
5	Patient origin	Native/Not native
6	Substance involved	Benzodiazepines/Buprenorphine/Clomethiazole/Codeine/Dextromethorphan/Fentanyl and related compounds/Methadone/Methylphenidate/Misoprostol/Modafinil/Tramadol/Others
7	Drug request	Does not require a prescription/Requested with prescription/Requested without prescription/Probably forged prescription
8	Intimidation	Yes/No
9	Frequent request	Yes/No
10	Pharmacist management	Supplied/Not supplied
11	Why do you supply the medicine?	
12	Observations	

## Data Availability

The datasets that support the findings of this study are available from the first author (M.P.) upon reasonable written request.
